# Evidence for non-selective response inhibition in uncertain contexts revealed by combined meta-analysis and Bayesian analysis of fMRI data

**DOI:** 10.1038/s41598-022-14221-x

**Published:** 2022-06-16

**Authors:** Ruslan Masharipov, Alexander Korotkov, Svyatoslav Medvedev, Maxim Kireev

**Affiliations:** grid.4886.2 0000 0001 2192 9124 N.P. Bechtereva Institute of the Human Brain, Russian Academy of Sciences, Academika Pavlova Street 9, St. Petersburg, 197376 Russia

**Keywords:** Cognitive neuroscience, Motor control

## Abstract

Response inhibition is typically considered a brain mechanism selectively triggered by particular “inhibitory” stimuli or events. Based on recent research, an alternative non-selective mechanism was proposed by several authors. Presumably, the inhibitory brain activity may be triggered not only by the presentation of “inhibitory” stimuli but also by any imperative stimuli, including Go stimuli, when the context is uncertain. Earlier support for this notion was mainly based on the absence of a significant difference between neural activity evoked by equiprobable Go and NoGo stimuli. Equiprobable Go/NoGo design with a simple response time task limits potential confounds between response inhibition and accompanying cognitive processes while not preventing prepotent automaticity. However, previous neuroimaging studies used classical null hypothesis significance testing, making it impossible to accept the null hypothesis. Therefore, the current research aimed to provide evidence for the practical equivalence of neuronal activity in the Go and NoGo trials using Bayesian analysis of functional magnetic resonance imaging (fMRI) data. Thirty-four healthy participants performed a cued Go/NoGo task with an equiprobable presentation of Go and NoGo stimuli. To independently localize brain areas associated with response inhibition in similar experimental conditions, we performed a meta-analysis of fMRI studies using equal-probability Go/NoGo tasks. As a result, we observed overlap between response inhibition areas and areas that demonstrate the practical equivalence of neuronal activity located in the right dorsolateral prefrontal cortex, parietal cortex, premotor cortex, and left inferior frontal gyrus. Thus, obtained results favour the existence of non-selective response inhibition, which can act in settings of contextual uncertainty induced by the equal probability of Go and NoGo stimuli.

## Introduction

Response inhibition is the ability to suppress inappropriate, automatic, reflexive, or habitual prepotent responses to produce a controlled goal-directed response^1,2^. It is generally accepted in the literature that response inhibition works in close relation to other processes associated with cognitive control, such as working memory, voluntary attention, conflict monitoring, and action selection^3–7^. Moreover, response inhibition is thought to represent a multifaceted phenomenon rather than a unitary brain mechanism. A distinction is made between action cancellation and action restraint^7–9^ as well as between reactive and proactive response inhibition mechanisms^10–14^.

According to the conventional view, the response inhibition process is selectively triggered by “inhibitory” stimuli that result in increased neuronal activity in brain structures responsible for inhibitory control^15–18^. However, in several cases, the concept of selective response inhibition fails to explain observed behavioural and neurophysiological phenomena. Manipulating the probability of the occurrence of “inhibitory” stimuli and the subjects’ awareness of the probability of the appearance may slow down the motor response^10,19–25^. Moreover, when necessary to rapidly suppress a specific action, the action is inhibited along with all other potential actions. That is, such inhibition can affect the entire motor system^26–32^. In an attempt to explain the effects mentioned above, several authors proposed the concept of non-selective (“global”) response inhibition^12,18,33,34^. It is thought that these non-selective mechanisms serve to prevent inappropriate or premature responses at the expense of the speed of execution of correct actions. First, response inhibition mechanisms may non-selectively inhibit all potential responses to further selectively execute an appropriate response (non-selective inhibition of multiple concurrent motor responses). Second, inhibition may be triggered not only by the presentation of “inhibitory” stimuli but also by the occurrence of any imperative stimuli instructing on the necessity to suppress or execute a prepared action (non-selectivity of inhibitory stimulus perception). In the present work, we consider the latter mechanism.

A tentative neurophysiological model of the non-selective or “global” response inhibition involved in the resolution of interference between several competing response options was proposed by Frank^33^ and included the cortico-subthalamic “hyper-direct” pathway^35,36^ which is capable of rapidly and non-selectively suppress all potential response options. As the research area developed, it was hypothesized that the model might be applied not only to tasks with multiple concurrent response options but also to simpler tasks where the subject has to choose between executing and refraining from an action^18,34^. The authors used a cued equiprobable Go/NoGo task, wherein a preparatory cue stimulus indicated the probability of a NoGo stimulus occurrence. A simple equiprobable Go/NoGo was chosen instead of a complex Go/NoGo task to limit confounds between response inhibition and the accompanying cognitive processes^6^. Complex Go/NoGo tasks usually utilize the low probability of NoGo stimulus, difficulties in identifying NoGo signals, high attentional or working memory loads. Although one of the possible ways to build up a prepotent response tendency is to bias the Go/NoGo probabilities in favour of Go stimuli, it is not necessary when the design involves a simple speeded reaction time task with a single response and reduces the complexity of the identification of Go and NoGo signals to a single bit of information^18^. In these conditions, a stimulus that does not require a response elicits subthreshold automatic motor activations that do not become overt because they are counteracted by fast automatic response inhibition^20,37–40^.

Within the framework of the selective inhibition model, the inhibition process would only be triggered by the identification of the NoGo stimulus. According to the hypothesis of non-selective inhibition, when the context is uncertain (equal probability of NoGo and Go stimuli), the need for response inhibition arises for both NoGo and Go trials. Experimental assessment of the hypotheses revealed no statistically significant differences between the NoGo and Go trials in both the amplitude of the early components of event-related potentials (ERP)^34^ and the level of neuronal activity measured by functional magnetic resonance imaging (fMRI)^18^. At the same time, a significant difference in early ERP amplitudes (peaked at 170 ms) was found in uncertain equiprobable NoGo and Go trials compared to certain Go-control trials, where no inhibition is required^34^. The authors considered this fact as evidence for the presence of a “non-selective” inhibitory mechanism that is not specific to the processing of NoGo signals. The analysis of early ERP components suggests that non-selective inhibition may blindly suppress any automatic response when the context is uncertain, acting as a gating mechanism controlling the initiation of a prepared, prepotent response.

However, a critical limitation of the above-mentioned studies was that the authors could not accept the null hypothesis, so they did not provide direct proof of the practical equivalence of neuronal activity level between Go and NoGo trials in inhibition-related brain structures. Indeed, within the framework of classical (frequentist) null hypothesis significance testing (NHST), we cannot accept the null hypothesis based on the absence of a significant difference. We can only reject it. Thus, the question of experimental support for non-selective response inhibition remains unanswered, and answering this question requires overcoming the methodological limitation of NHST, which is possible using Bayesian statistics^41,42^.

Therefore, the present study aimed to verify the non-selective response inhibition hypothesis by using the fMRI data from an equiprobable Go/NoGo task. Based on the results of previous studies, it may be suggested that if the hypothesis on the non-selectivity of inhibition is correct, then the brain structures responsible for response inhibition will demonstrate practically equivalent levels of neuronal activity in equiprobable Go and NoGo trials. Bayesian parameter inference (BPI) was applied to assess this prediction. Instead, if the hypothesis on the selectivity of response inhibition is correct, then activation of inhibition-related brain structures will be observed in the NoGo conditions compared to the Go conditions.

## Methods

### A meta-analysis of fMRI studies using equal probability Go/NoGo tasks

Given that a practically equivalent level of neuronal activity can be identified not only for response inhibition-related structures, but also, for example, those related to sensory processing of visual stimuli, working memory, and attention, we conducted a meta-analysis of fMRI studies to independently localize brain structures associated with response inhibition. Studies using equal probability Go/NoGo tasks were selected for meta-analysis. We searched for studies that, similar to the investigation by Albares et al.^34^ and Criaud et al.^18^, compared neuronal activity in the condition of equiprobable presentation of Go and NoGo stimuli with the control Go condition, in which the subject did not need to inhibit the prepared action. We hypothesized that inhibition would be non-selectively elicited by both equiprobable Go and NoGo-stimuli when the context is uncertain. To test the non-selective inhibition hypothesis, we considered similar Go/NoGo tasks with an equiprobable presentation of Go and NoGo-stimuli. We did not consider Go/NoGo tasks with rare NoGo-stimuli, since studies using them have previously shown the involvement of selective response inhibition^15–17^.

**Figure 1:**
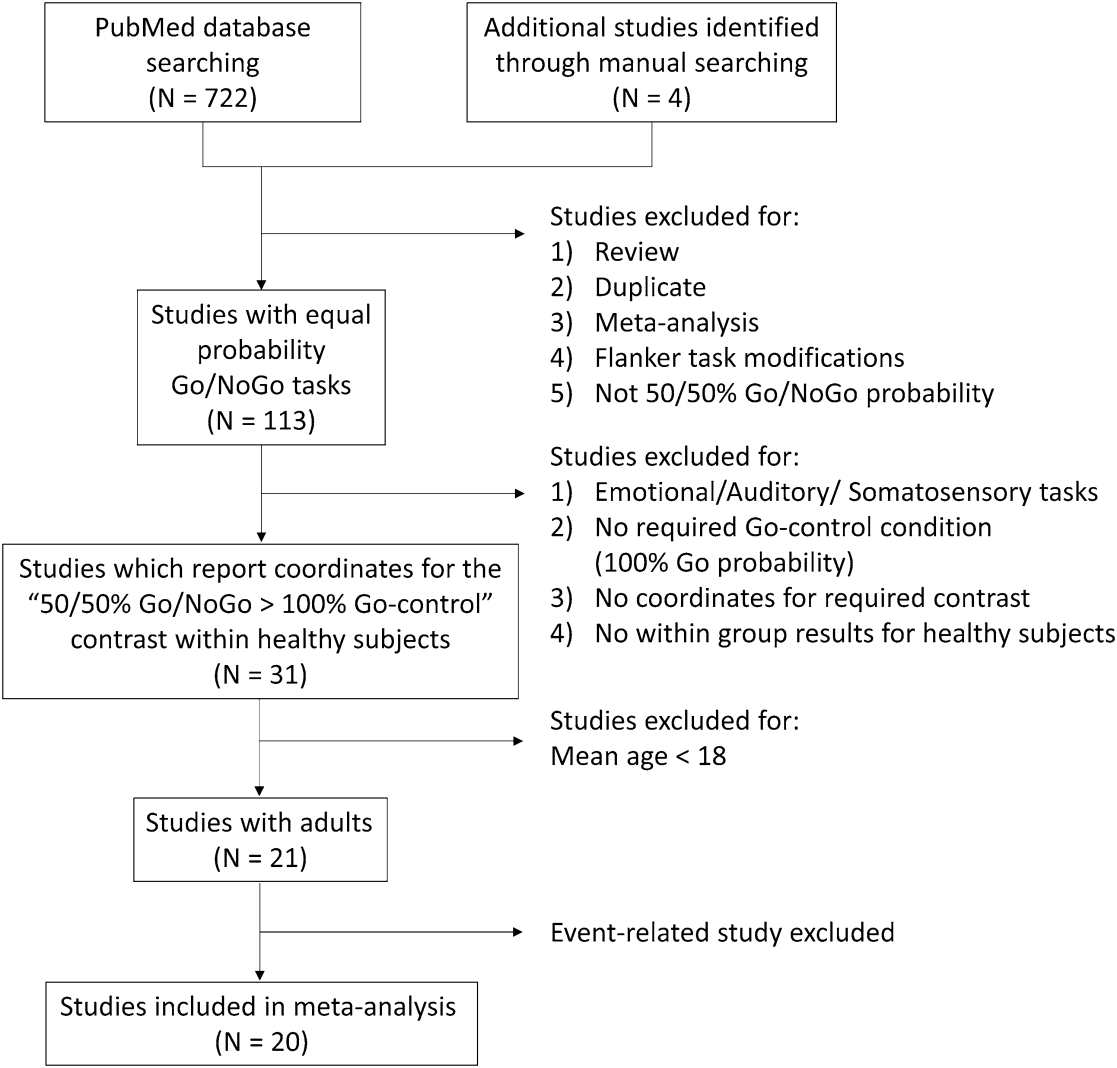
Flow chart of study selection in the meta-analysis.

We searched for these studies in the PubMed database in the period from 01/01/2000 to 15/07/2019 using the following keywords: “((fmri) OR (functional magnetic resonance)) AND ((nogo) OR (no-go)).” Four additional studies were identified through manual searches. As a result, 726 papers were identified. At the first stage of selection, we excluded reviews, meta-analyses and papers repeatedly reporting the results of fMRI studies (see the flow chart of analysis in Fig. 1).

Studies dealing with Flanker task modifications of Go/NoGo task or based on unequal probability of appearance of Go and NoGo trials were also excluded from the analysis. As a result, 593 papers were excluded. The task designs of the remaining 113 papers provided for equal probability Go and NoGo stimuli presentation. At the next step, 82 of the papers were excluded based on the following criteria: auditory and sensorimotor Go/NoGo tasks were used; only emotion-laden task conditions were used (emotionally neutral conditions were either absent or not considered separately); required Go-control condition (100% probability of the Go stimulus presentation) was not used; the coordinates for the contrast of interest “50/50% Go/NoGo > 100% Go-control” within a group of healthy subjects were not reported. In 11 out of the 31 remaining studies, healthy volunteer subjects under the age of 18 (children and adolescents) were studied. These articles were also excluded from the analysis because this study focused on brain activity of healthy, adult subjects. In all remaining studies except one, only block designs were used. To make our sample more homogeneous, we excluded the only eligible study with an event-related design^18^. The final meta-analysis included 20 studies (452 healthy subjects, mean age 29 years) with a total of 210 foci (for more details, see the “Supplementary materials”, Table S1). All coordinates were converted into Montreal Neurological Institute (MNI) space using the Lancaster transform^43^. The number of studies included in the current meta-analysis meets the minimum recommended number of 17–20 studies^44^.

Coordinate-based meta-analysis (CBMA) was performed using the random-effects activation likelihood estimation (ALE) algorithm^45–48^ implemented in the GingerALE 3.0.2 software (http://brainmap.org/ale) and Seed-based *d* Mapping with Permutation of Subject Images (SDM-PSI) algorithm^49^ implemented in the SDM-PSI 6.22 software (http://www.sdmproject.com). Two different CBMA algorithms were used for the cross-method validation.

The ALE algorithm assesses the spatial convergence between neuroimaging studies by modelling spatial uncertainty of activation foci using isotropic three-dimensional Gaussian probability functions with the full-width at half-maximum (FWHM) inversely related to the square root of the sample size of the original study^42^. An ALE map is obtained by computing the union of activation probabilities across studies for each voxel and tested against a null distribution of random spatial convergence between studies^43^. The empirical validations^50,51^ showed that the optimal FWHM largely depends on the data and may be substantially larger than proposed in^45^. The spatial smoothness of our fMRI data after preprocessing was 13.5 mm, as estimated by the SPM12 (Statistical parametric mapping) software package (http://www.fil.ion.ucl.ac.uk/spm). Since our objective was to find an overlap between the results of the meta-analysis and the Bayesian analysis of the obtained fMRI data, we used an additional FWHM of 4 mm for the ALE meta-analysis. It allowed us to achieve a median kernel FWHM of 13.5 mm. We used a cluster-level extend threshold of 0.05 corrected for family-wise error (FWE) with an uncorrected cluster-forming threshold of 0.001 (5000 threshold permutations) recommended for the ALE analysis^44^.

The alternative CBMA algorithm, SDM-PSI, considers not only the coordinates of the activation foci, but also the effect sizes. The SDM-PSI algorithm recreates 3D effect size images for each study using t-values of the peak coordinates and anisotropic kernels^52^. It has been shown that the recreation substantially improved by fully anisotropic kernels and did not depend on the FWHM^52^. We performed the SDM-PSI meta-analysis using the same coordinates as for the ALE meta-analysis adding peak t-values (z-values were converted to t-values). We used the default kernel (α = 1, FWHM = 20 mm) and threshold (threshold-free cluster enhancement FWE-corrected threshold of 0.05 with 5000 permutations) recommended by SDM developers^49,52^.

### Subjects

The recruitment of subjects for the present fMRI study was carried out in two stages. At the first stage, the sample size was 20 subjects (16 women, aged (mean ± SD) 23.9 ± 4.6). To protect from the potential impact of relatively small sample size on the observed null-effects a retrospective power analysis was carried out, which suggested increasing the sample size up to 34 subjects. Therefore, the final sample consisted of 34 healthy, right-handed volunteer subjects (24 women, aged 25.9 ± 5.2). An Oldfield test was used to determine the dominant arm^53^. The subject volunteers signed a written informed consent to participate in the study and were paid for their participation. All procedures were performed in accordance with the Declaration of Helsinki and were approved by the Ethics Committee of the N.P. Bechtereva Institute of the Human Brain of the Russian Academy of Sciences.

### Power analysis

To perform power analysis, we used the Consortium for Neuropsychiatric Phenomic LA5c dataset^54,55^ as it contains fMRI data from a relatively large cohort (N = 115) of healthy subjects performing a stop-signal task (see “Supplementary materials” for details). This classical inhibitory paradigm models a situation in which inhibitory brain activity is selectively triggered by infrequent inhibitory stimuli (stop signal) but not by Go stimuli^18^. The mean effect sizes for the selective inhibition contrast (“Correct-Stop > Go” contrast, see Fig. S1) were estimated within the cortical brain regions revealed by the current meta-analysis. They ranged from 0.57 to 1.12 Cohen’s d. Power analysis was performed using GPower 3.1.9.7^56^. It indicated that the sample size of 34 subjects would be sufficient to detect the minimum expected selective inhibition effect (d = 0.57, two-tailed one-sample test, alpha = 0.05) with a power of 0.9 (see Fig. S2). The power of 0.8 or 0.9 is commonly used for sample size calculations^57^.

However, it is worth noting that obtaining non-significant results with high retrospective power (based on an independent dataset) or high prospective power would not provide direct evidence for the null hypothesis^58^). To provide evidence for the null hypothesis, Bayesian inference or frequentist equivalence testing should be used^41,42,59,60^. Accordingly, in the current study, all conclusions on the non-selective response inhibition were made only on the basis of the Bayesian analysis and its overlap with the meta-analysis. Results for the classical NHST were added only to illustrate how the more familiar frequentist inference relates to Bayesian inference.

### Experimental task and study procedure

**Figure 2:**
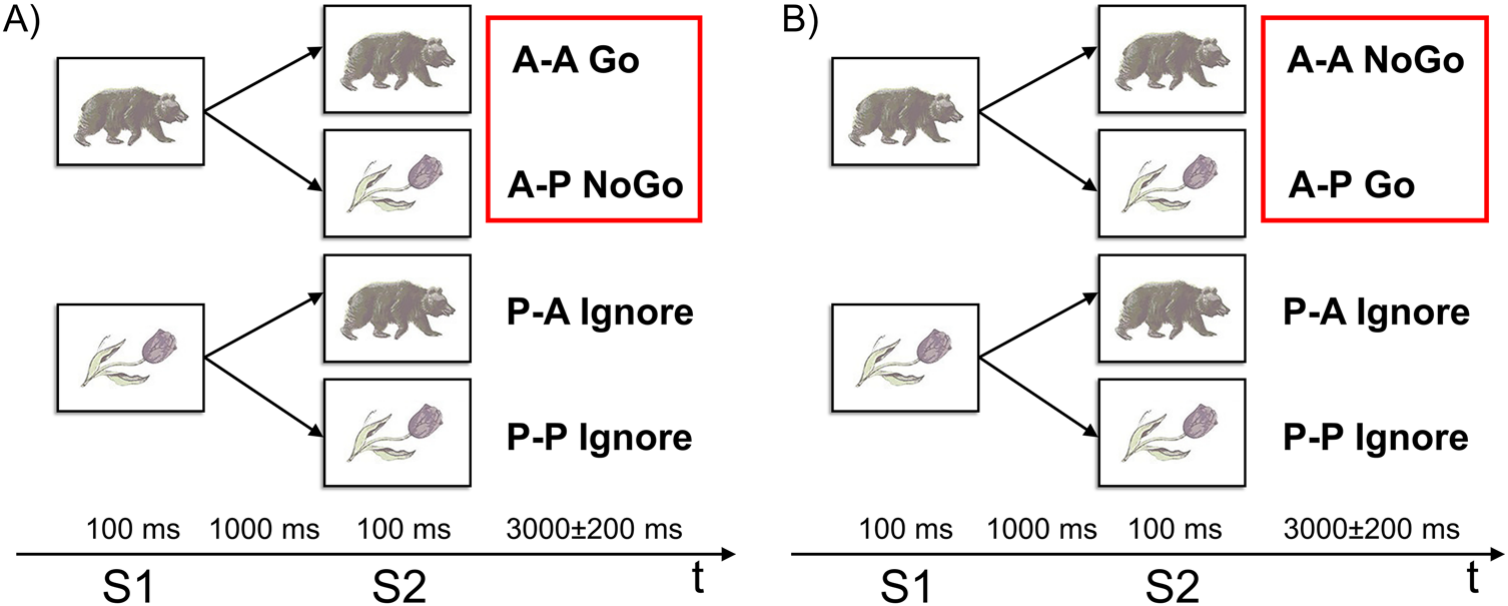
Experimental design of the Go/NoGo task. S1—first stimulus (preparatory cue). S2—second stimulus (imperative stimulus). A—images of animals, P—images of plants. (**A**) First experiment: “A-A Go”, “A-P NoGo”. (**B**) Second experiment: “A-P Go”, “A-A NoGo”. Red boxes highlight trials that were compared to test the hypothesis of selective and non-selective response inhibition.

We used a paired stimulus modification of the Go/NoGo task (see Fig. 2)^61^. This task was originally developed to dissociate cognitive processes, such as response inhibition, conflict monitoring, sensory mismatch and category discrimination^62,63^. Given that imperative Go and NoGo stimuli were presented in an equiprobable manner this modification of the Go/NoGo task was used for the purposes of the current study. Each trial consisted of the two consequently presented stimuli. The first preparatory cue stimulus warned the subjects on the presentation of the second, imperative stimulus, or indicated no need for any response to the second stimulus. The study included two variants of the experiment’s instructions. According to the first instruction (see Fig. 2A), the subject should press the response button as soon as possible upon presentation of the pair of images “animal-animal” (“A-A Go” trials) and refrain from acting upon presentation of the pair “animal-plant” (“A-P NoGo” trials). According to the instructions of the second experiment (see Fig. 2B), the subject acts after presentation of the pair “animal-plant” (“A-P Go” trials) and suppresses an action upon presentation of the pair “animal-animal” (“A-A NoGo” trials). The subjects were familiarized with the task just before scanning to ensure they understood the instructions. Additionally, before each fMRI session, the subjects were reminded of the need to react to Go stimuli as quickly as possible while refraining from reacting to NoGo stimuli.

In both experiments, if the first stimulus presented was an image of a “plant,” subjects do not need to take any actions in response to the presentation of any second stimuli of a trial. In such a trial, the subject should ignore the second stimulus and wait for the next pair of stimuli (“P-A Ignore” and “P-P Ignore” trials). Accordingly, it was assumed that there is no inhibition of the prepared action in “Ignore” conditions. The “Ignore” trials were included in the adopted Go/NoGo task to assess preparatory brain activity, such as preparing to receive a relevant stimulus (attentional set) and preparing to make a movement (motor set), by comparing them to Go and NoGo trials. However, the issue related to the preparatory processes is beyond the scope of the present study. Fifty pairs of each type of stimuli were randomly presented in each experiment. The order of following the instructions was counterbalanced among the subjects. Two variants of the present Go/NoGo task allowed us to control the differences in the load on working memory between A-P/A-A–Go/NoGo stimuli^63^.

The fMRI data obtained using this experimental task allowed us to test the hypothesis on the *non-selectivity* of response inhibition in the current study, since a similar task design was previously used for the same purpose by Criaud et al.^18^. The imperative Go and NoGo stimuli were presented after the preparatory stimulus with equal probability, as reported in event-related studies by^18,34,61,64^. To build up prepotent tendency to react, subjects were instructed to press the button with their right thumb as quickly as possible. This is a case of a simple speeded reaction time task with a single response and only one bit of information. It is known that these conditions elicit subthreshold automatic motor activations^18^. According to G.A. Miller: “One bit of information is the amount of information that we need to make a decision between two equally likely alternatives”^65^. Here, it refers to discrimination between “animal” and “plant” stimuli.

Such an equally probable presentation of imperative stimuli provides several advantages. First, it minimizes the difference in cognitive load between Go and NoGo conditions arising from task complexity^6,18^. Second, such a design enables the exclusion of effects associated with a low frequency of the NoGo stimuli presentation (“oddball” effects) confounding the effect of inhibition^64^. Third, it creates maximum uncertainty regarding the probability of the presentation of an imperative stimulus, thus minimizes the conflict between two response types, making it possible to distinguish between error monitoring and conflict resolution processes on the one hand and response inhibition processes on the other^66^.

In total, 100 NoGo trials, 100 Go trials, 100 P-A Ignore trials, and 100 P-P Ignore trials were presented over two experimental sessions. In the absence of stimulation, a fixation cross was displayed in the centre of the screen. The stimuli were presented for 100 ms, and the interstimulus interval was 1000 ms. The intertrial interval jittered from 2800 to 3200 ms with an increment step of 100 ms. Additionally, to improve design efficiency, 100 zero events (fixation crosses) were randomly inserted between the stimuli pairs (trials), and their duration jittered from 3000 to 5000 ms with an increment size of 500 ms. The action to be performed consisted of pressing a button with the right thumb. The duration of one task session was 17.5 min. Before starting the fMRI study, the subjects performed a training task. The Invivo’s Eloquence fMRI System (Invivo, Orlando, FL, USA) was used to deliver the stimuli, synchronize with fMRI acquisition, and record reaction times of subjects’ bottom pressing. The task presentation sequence and all temporal parameters of the stimuli presentation were programmed using the E-prime 2.0 software package (Psychology Software Tools Inc., Pittsburgh, PA, USA).

### Image acquisition

A Philips Achieva 3.0 Tesla scanner (Philips Medical Systems, Best, Netherlands) was used for the study. The structural T1-images were registered with the following parameters: field of view (FOV)—240 × 240 mm, repetition time (TR)—25 ms, echo time (TE)—2.2 ms, 130 axial slices with a thickness of 1 mm and pixel size of 1 × 1 mm, flip angle—30°. For the registration of T2*-images, a single-pulse echo planar imaging (EPI) sequence was used. The period of data registration from 31 axial slices was 2 s (TR = 2 s, TE = 35 ms). The following parameters were employed: FOV—200 × 186 mm, flip angle—90°, voxel size—3 × 3x3 mm. Two dummy scans were performed prior to each session. To minimize head movements, we used an MR-compatible soft cervical collar and foam padding.

### Preprocessing of fMRI images

Image preprocessing included the following: realignment to the first image of the session, slice time correction, co-registration, segmentation, normalization to an MNI template, and spatial smoothing (8 mm FWHM). Preprocessing and statistical analyses of the images were performed using an SPM12. To assess head motion during the scanning, we calculated the framewise displacement (FD) as the sum of the absolute values of the derivatives of the six realignment parameters^67^. The mean FD across two runs ranged from 0.07 to 0.36 mm (group mean ± standard deviation was 0.19 ± 0.07 mm). The average percentage of time points with FD over 0.9 mm was 1 ± 2%. We used all time points for the analysis since all subjects had > 90% volumes under the FD threshold of 0.9 mm^68,69^.

### Lateralized BOLD response analysis

One of the electrophysiological indicators of overt and covert motor activations is the Lateralized Readiness Potential (LRP)^70^. The equiprobable Go/NoGo study by Hong et al.^71^ showed an increase of the right-hand target-related LRP (“C3 minus C4” ERP difference) in NoGo trials compared to Ignore trials, which was considered as evidence of a prepotent response tendency. The fMRI analogue of LRP is the Lateralized BOLD response (LBR)^72,73^. Therefore, we used the LBR to index prepotent motor activations under task settings of the present study. The right-hand LBR was calculated as the mean BOLD signal in the left sensorimotor cortex (SM) minus the right SM cortex (“L SM minus R SM” BOLD difference). BOLD signal was estimated within 0.5 s time bins from the target onset time using finite impulse response models (time window from 1 to 12 s). Regions of interest (ROIs) located in the SM cortex were defined as the overlap between anatomical and a priori functional masks^42,74^. Functional masks were obtained using the Neurosynth platform for an automatic meta-analysis (https://neurosynth.org). We used a uniformity test with a default false discovery rate (FDR) corrected p < 0.01 threshold for the “motor” term (2565 studies). To obtain anatomical masks, we used voxels with the maximum probability for “precentral gyrus” and “precentral gyrus” labels according to the probabilistic Harvard–Oxford atlas^75^. ROI analysis was performed using one-sample and paired-sample t-tests with FDR correction for multiple comparisons.

### Statistical analysis of fMRI data

The first level of analysis was conducted using frequentist parameter estimation. The second level of analysis was performed using both the frequentist and Bayesian parameter estimation^76–78^. In general, using Bayesian analysis on the second level does not presuppose Bayesian parameter estimation at the first level of analysis. One can combine computationally less demanding frequentist parameter estimation for single subjects with Bayesian estimation and inference at the group level^78^. Onset times of second stimuli presentation (separately for “A-A Go”, “A-P NoGo”, “P-A Ignore Exp1”, “P-P Ignore Exp1”, and also “A-P Go”, “A-A NoGo”, “P-A Ignore Exp2”, “P-P Ignore Exp2”), erroneous button pressing, and missing the responding in Go trials were used to create regressors of the general linear model (GLM) for each subject. Events were impulse responses with a duration of zero convolved with the canonical haemodynamic response function (HRF). We also performed an additional analysis with the temporal and dispersion derivatives for all task regressors to take into account the unknown BOLD delay. Low-frequency drift was removed by temporal high-pass filtering with a cut-off frequency of 1/128 Hz. Six head motion parameters were included in the GLM as nuisance regressors to account for the movement artefacts^79^. Beta coefficients reflecting an increase in the blood oxygenation level-dependent (BOLD) signal in the experimental condition relative to the implicit baseline were scaled to percent signal change (PSC) following the procedure recommended in [Ref.^80^, p.186]. To this end, the beta coefficients for each condition were divided by the mean value of the beta coefficients for the constant term and multiplied by 100 and a scaling factor (SF) needed so that the peak of an isolated BOLD response is equal to one (SF = 0.21). Two linear contrasts of scaled beta coefficients were calculated: (1) 0.5 × [“A-P NoGo” + “A-A NoGo”]—0.5 × [“A-P Go” + “A-A Go”] *(the “NoGo vs. Go” comparison);* (2) 0.25 × [“A-P NoGo” + “A-A NoGo” + “A-P Go” + “A-A Go”]—0.25 × [“P-A Ignore Exp1” + “P-P Ignore Exp1” + “P-A Ignore Exp2” + “P-P Ignore Exp2”] *(the “Go* + *NoGo vs. Ignore” comparison).* The sum of positive contrast weights was equal to one. The contrasts from 34 subjects were used as variables to verify the hypotheses on selective and non-selective response inhibition at the second level of analysis. Only grey matter voxels were included in the second-level analysis, as we did not expect to detect the BOLD signal changes in white matter associated with the Go/NoGo task performance. To that end, a mask was created based on the segmentation of each subject’s structural T1-images.

### Verification of the hypotheses on selective and non-selective response inhibition

As in previous studies employing equiprobable Go/NoGo task with a single prepotent motor response, in the current study, non-selectivity refers to the perceptual decision mechanisms involved in the detection, discrimination, or identification of sensory stimuli^18,34^. In particular, we did not consider non-selectivity related to decision mechanisms that involve the selection between multiple alternative responses, which confound response inhibition processes in choice reaction time tasks^6,18^. The former type of non-selectivity implies that any imperative stimuli (both Go and NoGo stimuli) would trigger response inhibition when the context is uncertain because of equal probability of Go and NoGo stimuli. The latter type of non-selectivity, usually called “global” inhibition, implies that inhibition affects all alternative responses, including the selected response^27^.

We tested the hypotheses on selective and non-selective response inhibition using the “NoGo vs. Go” comparison. In the case of the *selectivity* of response inhibition, we expect to find a selective increase in the neuronal activity in response to the presentation of NoGo stimuli compared to Go stimuli (“NoGo > Go”). In the case of the *non-selectivity* of response inhibition, we expect to find a practically equivalent increase in the neuronal activity in response to the presentation of both NoGo and Go stimuli (“NoGo = Go”) in the brain areas related to the response inhibition, which were independently localized by the current meta-analysis (“50/50% Go/NoGo blocks > 100% Go-control blocks”). Additionally, we used the “Go + NoGo vs. Ignore” comparison to distinguish between the brain areas that are simply not activated in current task settings (“Go + NoGo = Ignore”) from the brain areas activated in equiprobable Go and NoGo trials compared to Ignore trials, where no inhibition is required (“Go + NoGo > Ignore”). Thus, to identify the non-selective response inhibition in the settings of contextual uncertainty induced by the equal probability of Go and NoGo stimuli, it was necessary to show a three-way overlap between (1) inhibitory-related brain areas according to the meta-analysis (“50/50% Go/NoGo blocks > 100% Go-control blocks”), (2) brain areas with practically equivalent neuronal activity in Go and NoGo trials (“NoGo = Go”) and (3) brain areas activated in equiprobable Go and NoGo trials (“Go + NoGo > Ignore”). This conjunction analysis was performed by binarization and multiplication of thresholded images^81^.

To reveal brain areas with practically equivalent neuronal activity in the Go and NoGo trials (“NoGo = Go”), one has to provide evidence for the null hypothesis. The classical NHST approach estimates the probability (p-value) of obtaining actual data’s best-fitting parameter value, *β*, or something more extreme under the null hypothesis that the experimental effect is *θ* = *cβ* = *0.* The null hypothesis can never be accepted using NHST because the p-value does not represent the probability of the null hypothesis, and the probability that an effect equals exactly zero is itself zero^76^. A non-significant result can be obtained in two cases^82^: (1) there is no effect, and our data are against the alternative hypothesis; or (2) our data are insufficient to distinguish alternative hypothesis from the null hypothesis, and we cannot confidently make any inference (low statistical power). When the result obtained is not significant, it is recommended to use frequentist equivalence testing^62^ or Bayesian inference^41,42,60,83^ to provide evidence for the null hypothesis. In the present study, Bayesian parameter inference (BPI) was used^42,60,77,83^.

The Bayesian approach estimates the posterior probability *(PP)* distribution of the effect *θ*, given the data using the likelihood and the prior knowledge. For the second-level Bayesian analysis, SPM12 implements the hierarchical parametric empirical Bayes approach with the global shrinkage prior^77^. It represents a prior belief that, on average, in the whole brain, there is no global experimental effect. If the posterior probability of the effect exceeding the effect size threshold, *γ,* is greater than the predefined probability threshold, *α* = 95%, then the hypothesis on the presence of “NoGo > Go” effect will be accepted (see Fig. S3A in the “Supplementary materials”):

$${PP}_{NoGo>Go}=p\left(\theta >\gamma |Data\right)\ge \alpha .$$

If the effect value falls within the interval [− *γ*; *γ*] with a probability of *α* = 0.95, then the hypothesis of the null “NoGo = Go” effect will be accepted, supporting the practical equivalence^60^ of the BOLD signal between the conditions compared (see Fig. S3B):

$${PP}_{NoGo=Go}=p\left(-\gamma <\theta <\gamma |Data\right)\ge \alpha .$$

The interval [− *y*; *y*] can be thought of as the neuronal “background noise level”^84^ or as a region of practical equivalence (ROPE) that expresses which effect size values (PSC) are equivalent to the null value for current practical purposes^41^.

The hypothesis on the presence of the “Go > NoGo” effect will be accepted if (see Fig. S3C):

$${PP}_{Go>NoGo}=p\left(\theta <-\gamma |Data\right)\ge \alpha .$$

If none of the above criteria are satisfied, the data in particular voxel are insufficient to distinguish the null hypothesis from the alternative hypothesis (“low-confidence” voxels, see Fig. S3D)^85^. For such voxels, it is impossible to make any inference confidently, so we need to increase the sample size or scanning time. In the current study, our conclusions were based only on voxels in which the effect size values (PSC) fell within or outside the interval [− *γ*; *γ*] with the *PP* > 0.95. Thus, for these voxels, the data obtained are sufficient to make an inference confidently. This decision rule is also known as the “ROPE-only” decision rule [^60^; see Fig. S3]. It was applied to the “NoGo vs. Go” and “Go + NoGo vs. Ignore” comparisons. BPI was performed using the *BayInf* toolbox based on SPM12 (https://github.com/Masharipov/Bayesian_inference)^42^. BPI based on the “ROPE-only” decision rule with a zero-effect size threshold corresponds to the false discovery rate correction for multiple comparisons within the NHST framework^77,86^. The group-level effect size threshold *γ* was set at one standard deviation of the prior variance of the contrast (prior SD_*θ*_), which is the default in SPM12^77,84^. Simulations showed that this threshold provides high sensitivity to both “activated” and “not activated” voxels while protecting against incorrect decisions^42^. BPI with the ES threshold of one prior SD_*θ*_ typically detect similar “activations” as classical NHST inference with a voxel-wise FWE-corrected threshold of 0.05^42^. To illustrate this, the BPI results were compared with the classical NHST results. For visualization purposes, the posterior probabilities were converted to the logarithmic posterior odds, LogOdds = log(*PP*/(1 − *PP*)). LogOdds > 3 correspond to *PP* > 0.95. Anatomical localization of clusters was identified using xjView toolbox (http://www.alivelearn.net/xjview).

### Sequential analyses

Since the statistical analysis was carried out twice, for the sample size N = 20 and N = 34, the question of “data peeking” should be addressed. “Data peeking” refers to the practice when a researcher periodically re-analyses sequentially obtained data and decides to stop data collection as soon as significant results are obtained^87^. Sequential analyses and optional stopping in the classical NHST framework substantially inflate the number of false positives and require special adjustments of the p-values along with the definition of the stopping rule based on the power analysis^88^. In contrast, Bayesian inference based on the posterior probabilities or Bayes factors does not depend on stopping intentions and does not necessarily require power analysis^41,89^. Therefore, the issue of “data peeking” may be mitigated by the usage of Bayesian statistics^90^.

Within the classical NHST framework, a statistically significant difference may be shown in any voxel with a sufficiently large sample size, even when the effect is trivial or has no practical significance^76^. Thus, with an unbounded increase of the data, the number of statistically significant voxels will approach 100% of the total number of voxels [see, for example^91^]. At the same time, Bayesian inference allows one to find not only “activated” voxels but also “null effect” voxels with a trivial difference (practical equivalence) and “low confidence” voxels for which obtained data are not sufficient. Increasing sample size using BPI will lead to the situation when the number of “activated” and “null effect” voxels reaches a plateau. When the plateau was reached, it is reasonable to stop the data collection. To illustrate how this relates to the current study, we provide an analysis of the dependencies between the sample size and the number of “activated”, “null effect,” and “low confidence” voxels for the “NoGo vs. Go” comparison. To plot these dependencies, the analysis was performed for the samples from 6 to 34 subjects with a step of 2 subjects (see the “Supplementary materials”). In total thirty random groups were sampled for each step.

## Results

### Meta-analysis

**Figure 3:**
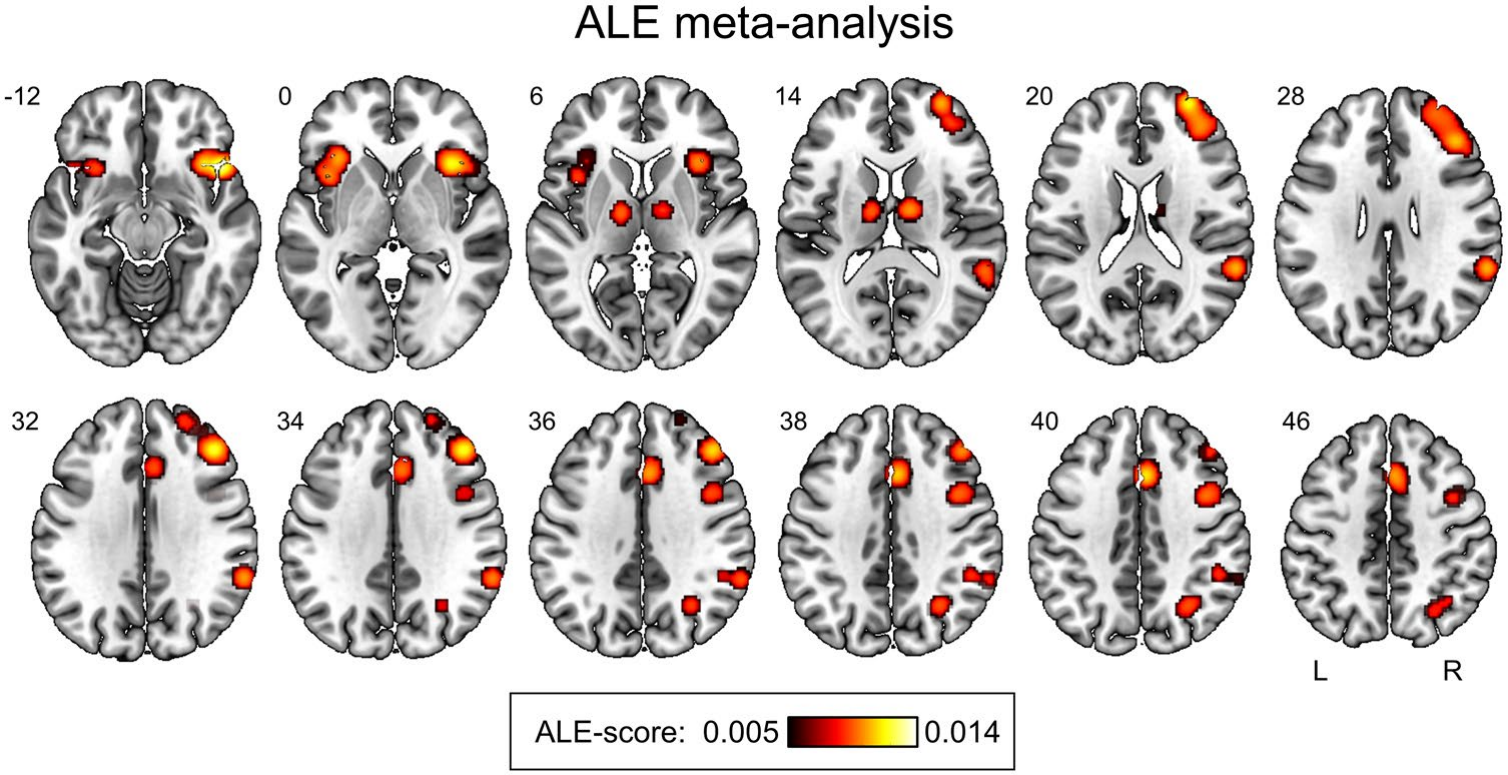
Results of the ALE meta-analysis. The result of the meta-analysis of 20 fMRI studies using equal probability Go/NoGo tasks (“50/50% Go/NoGo blocks > 100% Go-control blocks” contrast). Figure was created using MRIcroGL 1.2.2 software (https://www.nitrc.org/projects/mricrogl).

As a result of the ALE meta-analysis of 20 fMRI studies, we identified the brain structures that were characterized by increased neuronal activity demonstrated in settings of equal probability Go and NoGo stimuli presentation compared to the control Go conditions (“50/50% Go/NoGo blocks > 100% Go-control blocks” contrast): (1) right dorsolateral prefrontal cortex (DLPFC), (2) right inferior parietal lobule (IPL), (3) right temporoparietal junction (TPJ), (4) bilateral inferior frontal gyrus (IFG) and anterior insula (also known as anterior insula/frontal operculum (AIFO)), (5) right premotor cortex (PMC) and frontal eye field (FEF), (6) bilateral anterior cingulate cortex (ACC) and supplementary motor area (SMA), and (7) bilateral thalamus (see Fig. 3, Table 1). The SDM-PSI results were generally consistent with the ALE results, with the only difference being the absence of clusters in the subcortex (see Fig. S4 and Table S2 in the “Supplementary materials”). Therefore, below, we consider only clusters in the cerebral cortex.

**Table 1:** The result of the ALE meta-analysis of 20 fMRI studies using equal probability Go/NoGo tasks (“50/50% Go/NoGo blocks > 100% Go-control blocks” contrast).

No.	Cluster size (mm^3^)	Peak MNI-coordinate (mm)	Peak ALE-score	Anatomical localization (L—left, R—right hemisphere; BA—Brodmann area)
1	10,160	42 34 32	0.0114	R: DLPFC (middle frontal gyrus), BA 8,9
2	8368	46 18 − 10	0.0138	R: IFG/Anterior insula (AIFO), BA 13, 45, 47
3	4896	− 34 24 0	0.0088	L: IFG/Anterior insula (AIFO), BA 13, 44, 45, 47
4	4176	60 − 44 26	0.0090	R: TPJ (supramarginal gyrus and superior temporal gyrus), BA 22, 39, 40
5	3408	5 18 40	0.0103	R/L: SMA, preSMA, ACC, BA 6, 8, 24, 32
6	1664	12 − 8 10	0.0089	R: Thalamus
7	1584	42 6 40	0.0076	R: PMC, FEF (precentral gyrus, middle frontal gyrus), BA 6, 8, 9
8	1456	− 12 − 10 10	0.0084	L: Thalamus
9	1424	30 − 62 38	0.0071	R: IPL (angular gyrus), BA 7, 39

*DLPFC* dorsolateral prefrontal cortex, *IFG* inferior frontal gyrus, *AIFO* anterior insula/frontal operculum, *TPJ* temporoparietal junction, *SMA* supplementary motor area, *ACC* anterior cingulate cortex, *PMC* premotor cortex, *FEF* frontal eye field, *IPL* inferior parietal lobule.

### Behavioural data

In two fMRI sessions, the mean response omission in Go trials was 2.97 ± 3.92%. The mean of false alarms in NoGo trials was 0.35 ± 0.64%. The mean response time (RT) was 384 ± 60 ms.

### fMRI data

Neither classical NHST with the voxel-wise FWE-corrected threshold of 0.05 nor Bayesian inference applied in the present fMRI study revealed a significant increase in the neuronal activity in NoGo trials compared to the Go trials. No significant increases were revealed using a less conservative threshold-free cluster enhancement FWE-corrected threshold of 0.05 and a voxel-wise uncorrected threshold of 0.005. Additional analysis with the temporal and dispersion derivatives also did not reveal a significant “NoGo > Go” effect predicted by the hypothesis of selective response inhibition.

**Figure 4:**
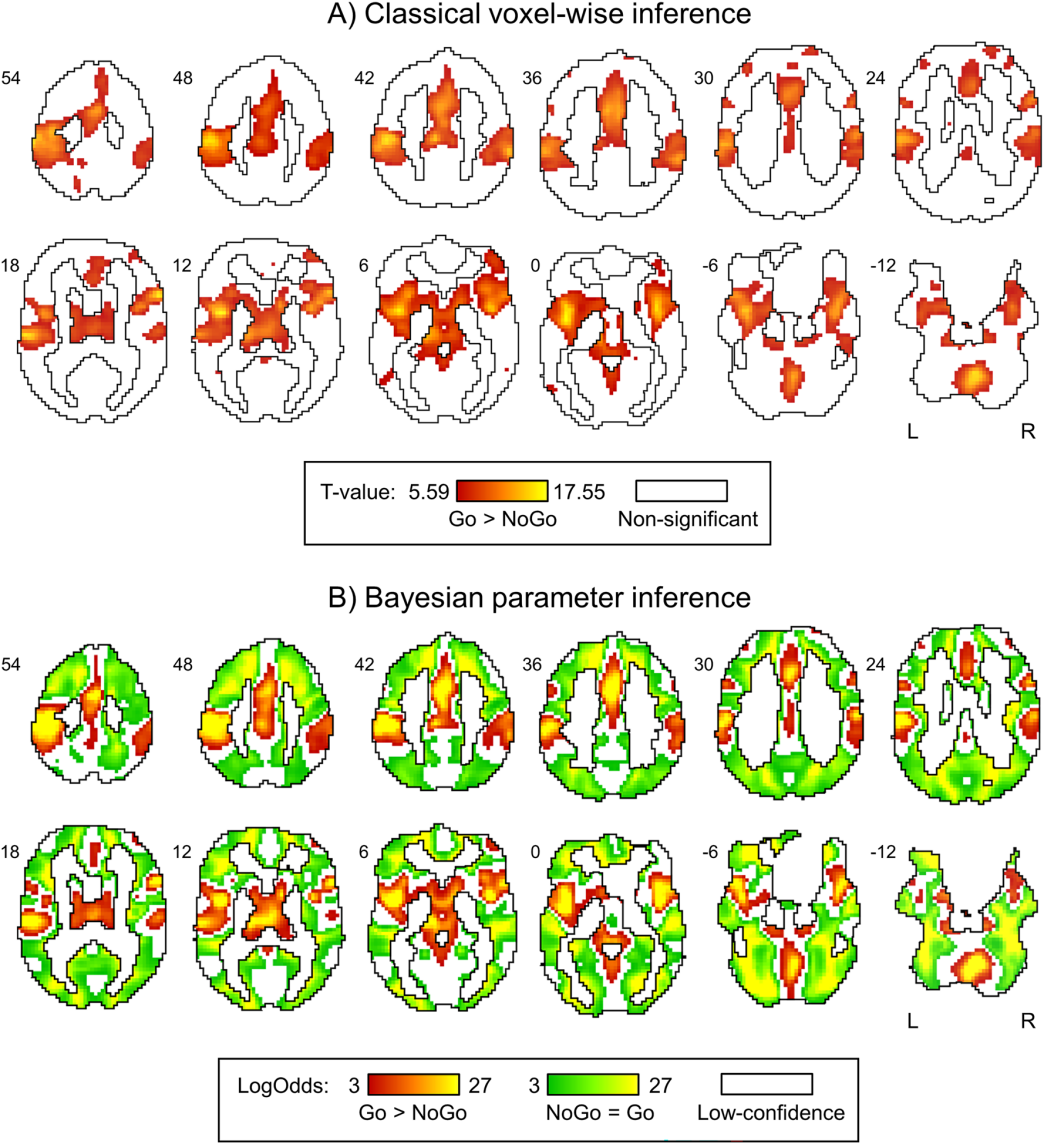
Results of classical voxel-wise and Bayesian parameter inference. (**A**) Classical NHST inference with an FWE-corrected voxel-wise threshold of 0.05. (**B**) BPI with the effect size threshold *γ* = 1 prior SD_*θ*_ = 0.1%, LogOdds > 3 (*PP* > 0.95). The “NoGo > Go” effect was not detected by any of the methods. Red colour depicts the “Go > NoGo” effect. Green colour depicts the “NoGo = Go” effect (practical equivalence of neuronal activity in Go and NoGo trials). White colour depicts (**A**) non-significant voxels and (**B**) “low-confidence” voxels. Figure was created using Mango 4.1 software (https://ric.uthscsa.edu/mango).

The reversed “Go > NoGo” contrast showed the expected motor activations in the pre- and postcentral gyrus, premotor cortex, supplementary motor area, subcortical regions and cerebellum (see Fig. 4 and Tables S3, Tables S4 in the “Supplementary materials”). Both statistical methods revealed similar activation patterns for the “Go > NoGo” effect (Dice coefficient = 0.8), demonstrating the consistency between the BPI with *γ* = 1 prior SD_*θ*_ and LogOdds > 3 *(PP* > 0.95) thresholding approach and NHST with the voxel-wise FWE-corrected threshold of 0.05.

At the same time, Bayesian analysis has made it possible to define brain structures with practically equivalent neuronal activity in Go and NoGo trials. The null “NoGo = Go” effect was revealed for a widely distributed set of regions throughout the entire brain surrounding clusters of activations revealed in the “Go > NoGo” contrast (see Fig. 4A). The “NoGo = Go” regions were separated from activation clusters by regions that consisted of “low-confidence” voxels^67^. For “low-confidence” voxels, the data obtained are insufficient to accept or reject the null hypothesis. The number of “low-confidence” voxels were decreased with increasing sample size (see Fig. S5 in the “Supplementary materials”). The largest gain in the number of “NoGo = Go” and “Go > NoGo” voxels can be noted from 6 to 20 subjects. After 20 subjects, the dependencies between the sample size and the number of “NoGo = Go” and “Go > NoGo” voxels reached a plateau. This is also confirmed by the similarity between the BPI results for the sample size of 20 and 34 subjects (Dice coefficient = 0.88 for the "NoGo = Go" effect, see also Fig. S6 and Table S5 in the “Supplementary materials”).

It should be noted that the “NoGo = Go” area cannot be reasonably split into several parts, such as activation clusters. Therefore, we limited ourselves to specifying the anatomic localization of brain areas where the “NoGo = Go” area overlaps with the clusters revealed by the current meta-analysis and brain areas activated by equiprobable Go and NoGo trials compared to Ignore trials (see “Results” for the “Go + NoGo > Ignore” contrast in the “Supplementary materials”, Fig. S7 and Table S6).

### LBR analysis

**Figure 5:**
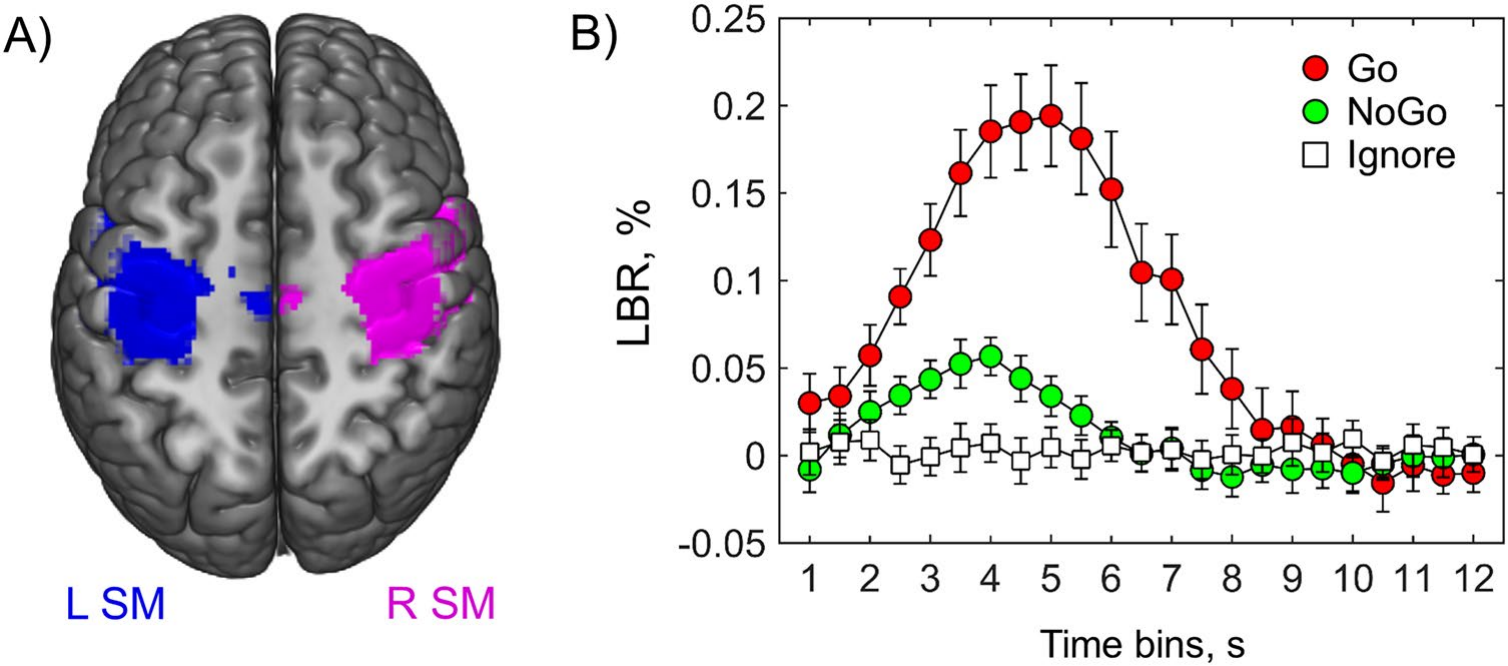
LBRs for the right hand (“L SM minus R SM” BOLD difference). (**A**) Sensorimotor ROIs in the left and right hemispheres (blue and violet color correspondingly). (**B**) Mean LBR (and 95% confidence intervals) for the Go, NoGo, and Ignore trials. Figure was created using MRIcroGL 1.2.2 software (https://www.nitrc.org/projects/mricrogl).

The right-hand LBRs were analysed to assess the prepotent motor tendency in equiprobable Go/NoGo task settings. We did not find a significant increase in LBR above zero for the Ignore condition in any of the analysed time bins (see Table S7 in the “Supplementary materials”). At the same time, we found a significant increase in LBR in the NoGo trials compared to the Ignore trials for time bins from 2.5 to 5.5 s (pFDR < 0.05). Therefore, the revealed increase in LBR supports the presence of prepotent motor activity in the NoGo trials (see Fig. 5).

### Conjunction analysis

**Figure 6:**
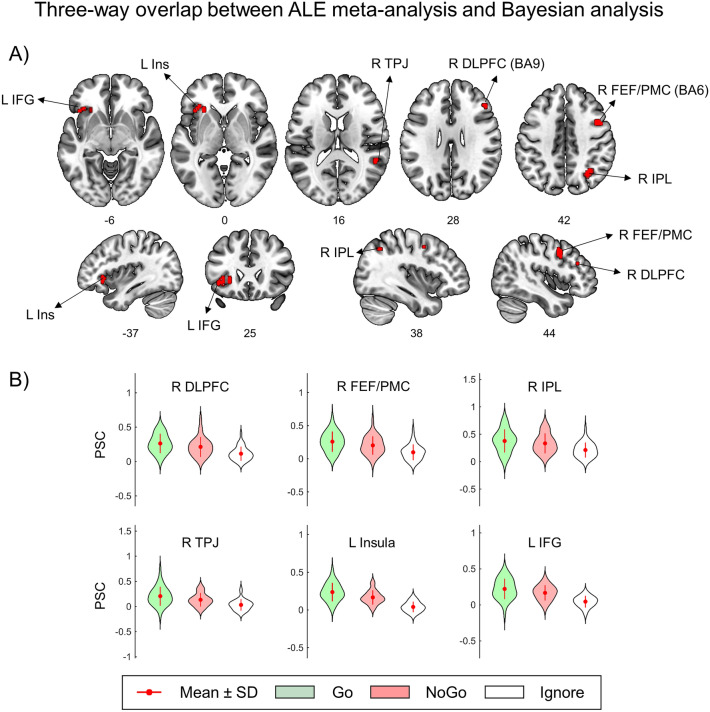
Three-way overlap between the ALE meta-analysis and Bayesian analysis. (**A**) Three-way overlap between (1) inhibitory-related brain areas according to the ALE meta-analysis (“50/50% Go/NoGo blocks > 100% Go-control blocks”), (2) brain areas with practically equivalent neuronal activity in Go and NoGo trials (“NoGo = Go”) and (3) brain areas activated in equiprobable Go and NoGo trials (“Go + NoGo > Ignore”). (**B**) Violin plots of the mean PSC in the revealed clusters. *DLPFC* dorsolateral prefrontal cortex, *FEF* frontal eye field, *PMC* premotor cortex, *IPL* inferior parietal lobule, *TPJ* temporoparietal junction, *Ins* insula, *IFG* inferior frontal gyrus, *BA* Brodmann area, *L* left, *R* right. Figure was created using MRIcroGL 1.2.2 software (https://www.nitrc.org/projects/mricrogl).

As it can be inferred from the revealed overlap between the results of the ALE meta-analysis and the results of the abovementioned Bayesian analysis, only a few of the brain structures demonstrated (1) increased activity in the “50/50% Go/NoGo blocks > 100% Go-control blocks” meta-analytical comparison, (2) the practical equivalence of the BOLD signal in the “NoGo vs. Go” comparison and (3) activation in Go and NoGo trials compared to Ignore trials. Three-way overlap was observed in (1) right DLPFC, (2) right IPL, (3) right TPJ, (4) left IFG and anterior insula (AIFO), (5) right PMC, and FEF (see Fig. 6, Table 2). The results of the three-way overlap obtained using the SDM-PSI meta-analysis were generally consistent with the ALE overlap (see Fig. S8 and Table S8 in the “Supplementary materials”). Two additional clusters were revealed using SDM-PSI meta-analysis: (1) left ACC/preSMA (BA 32) and (2) right anterior insula. In the discussion, we considered only those clusters that were found in three-way overlaps for both meta-analyses.

**Table 2:** Three-way overlap between the ALE meta-analysis and Bayesian analysis.

No.	Cluster size (mm^3^)	Centroid MNI-coordinates	Anatomical localization (L—left, R—right hemisphere; BA—Brodmann area)
1	486	30 − 60 48	R: IPL IPL
2	405	42 9 45	R: PMC, FEF, BA 6, 8 PMC, FEF, BA 6, 8
3	270	− 39 27 0	L: IFG IFG
4	135	− 27 27 0	L: Anterior insula Anterior insula
5	108	51 − 42 18	R: TPJ TPJ
6	81	45 33 28	R: DLPFC, BA 9 DLPFC, BA 9

*IPL* inferior parietal lobule, *PMC* premotor cortex, *FEF* frontal eye field, *IFG* inferior frontal gyrus, *TPJ* temporoparietal junction, *DLPFC* dorsolateral prefrontal cortex.

## Discussion

The results of the present study supported the hypothesis of non-selective response inhibition in the contextual uncertainty associated with the equiprobable presentation of Go and NoGo stimuli. Bayesian analysis of the obtained fMRI data provides evidence of the practical equivalence of neuronal activity evoked by Go and NoGo stimuli in several brain structures. According to the results of our meta-analysis, some of these brain structures, including the right DLPFC, IPL, and TPJ, the left IFG (AIFO), the right PMC, and FEF were associated with response inhibition. These structures were repeatedly found to be activated whenever the condition of equal probability presentation of Go and NoGo stimuli was compared with the control Go condition (“50/50% Go/NoGo blocks > 100% Go-control blocks” contrast). At the same time, we did not observe the selective response inhibition effect (“NoGo > Go”) in the current study. This finding is consistent with the data of several previous event-related fMRI studies that did not find significant activity increase in NoGo trials compared to Go trials under similar experimental conditions^18,64,92^, see also random-effects analysis within visual and audio modalities in^93^.

An important question is whether we did not able to observe selective response inhibition due to insufficient inhibitory load in our task setting. Previously, it was argued that some configurations of the Go/NoGo task may not reliably evoke prepotent motor activity and therefore their inhibition requirements are reduced^94^. The argument for this was supported by fronto-central P300 amplitude reduction observed in the slow-paced (i.e. stimulus-stimulus interval > 4 s) equiprobable Go/NoGo settings compared to settings with fast-paced rare NoGo trials. However, the study by Wessel^94^ did not consider the cue-target type paradigms, aimed to increase the likelihood of a prepotent motor response, as in the current study. Equiprobable cue-target Go/NoGo tasks have previously been successfully used to study prepotent response inhibition^18,34,61,64,71,95–98^. Moreover, the Wessel study^94^ considered fronto-central P300 as an index of inhibition of prepotent motor activity. However, some studies have suggested that the P300 component (and also the N200 component) is too late to explain the inhibition processes^34,40,99,100^. It was argued that a marker of inhibitory control should exhibit activity that precedes inhibitory effects at the level of the motor cortex or the effector muscles (about 150 ms after inhibitory stimuli presentation, see^34,99–106^.

Arguments on fast early inhibition were considered in a recent review by Diesburg and Wessel^107^ developing the “Pause-then-Cancel” model of motor inhibition in humans. This model was originally proposed by Schmidt and Berke^108^ based on subcortical rodent recordings. It assumes that inhibition of motor responses is a two-stage mechanism. In the first stage, any unexpected stimuli non-selectively and briefly delay or “Pause” actions. The “Pause” process has a global inhibitory effect on the motor system via the monosynaptic “hyperdirect” pathway from the cortex to the subthalamic nucleus and is similar to the “global inhibition” concept proposed by Frank^33^. In the second stage, if an unexpected stimulus is discriminated as inhibitory, a relatively slower inhibitory process fully “Cancels” action via the “indirect” pathway. Therefore, the “Pause” process “buys time” for stimulus discrimination and decision making. According to the “Pause-then-Cancel” model, the early transient suppression of corticospinal excitability and subthreshold EMG may be a reflection of the “Pause” process. Although Diesburg and Wessel^107^ have mainly focused on the stop-signal tasks, they also argued that their theoretical framework applies to motor inhibition in other paradigms, including the Go/NoGo tasks. The authors posit that the “Pause” process is part of a universal orienting response common to all salient, task-relevant events. This is in line with the hypothesis of the current study. When the context is uncertain because of the equiprobable presentation of Go and NoGo stimuli, any imperative Go or NoGo stimuli may non-selectively trigger the inhibitory process. Conceptually this is similar to the “Pause” process.

The presence of the prepotent response tendency in the current study was supported both by fast response times (⁓ 380 ms) and increased LBR in NoGo trials compared to Ignore trials (fee Fig. 5), in which no prepotent motor activity was expected. The former finding is closely match behavioural data from previous studies with similar equiprobable Go/NoGo design, where subjects were trained to react as fast as possible to create prepotent response tendency^18,34,109^. The later experimentally demonstrates the presence of covert prepotent motor activity in NoGo trials.

The other important question is how precisely observed activity can be associated with response inhibition per se in those brain regions in which the overlap was found between the meta-analysis and Bayesian analysis. It is well known that the frontoparietal structures (DLPFC, IPL, TPJ) are engaged not only in Go/NoGo tasks but also in experiments examining task switching, resolution of cognitive conflicts, working memory, and attention focusing (“multiple demand system”^110^; “task-general network”^111^; “extrinsic mode network”^112^. According to the “Pause-then-Cancel” model the non-selective inhibitory process (“Pause”) and attentional process may be indistinguishable, because attentional orienting to salient, task-related signals inherently accompanied by broad motor suppression^107^. Both processes are so fast and transient that their neural signatures may be near impossible to disentangle. Furthermore, attentional and inhibitory processes may rely on overlapping neural circuits.

However, several attempts have been made to dissociate inhibitory and attentional processes by using special modifications of Go/NoGo task that control for attentional loads. For example, the fMRI study by Chikazoe et al.^113^ showed greater activation to infrequent NoGo-stimuli than to infrequent Go-stimuli in the right posterior inferior frontal gyrus (pIFG). Since both NoGo and Go stimuli were infrequent, the authors associated pIFG activation with an inhibitory process. They also demonstrated attention-related activity in the right inferior frontal junction (anatomically close to pIFG) using infrequent Go stimuli vs. frequent Go stimuli contrast. The other fMRI study by Dodds et al.^114^ showed activation in the right IFG to NoGo trials without attentional shifting compared to Go trials with attentional shifting. Reverse contrast revealed activation in the left inferior parietal cortex. The authors suggested that the right IFG and the left inferior parietal cortex are preferentially activated during response inhibition and attentional shifting, respectively. Dissimilar results have been observed in the fMRI study by Meffert et al.^24^, which varied the frequency of Go and NoGo stimuli. They showed that the *left* IFG and dorsal pre-SMA were responsive to NoGo stimuli regardless of stimuli frequency. Meanwhile, a more ventral portion of pre-SMA and anterior insula showed greater activity to low frequency relative to higher frequency stimuli, regardless of response type. Finally, in the EEG studies by Hong et al.^115^ and Hong et al.^71^ the attention-related neural activity (P300 ERP and theta oscillations in the cingulate cortex) was dissociated from inhibitory-related activity by comparing between attended-NoGo and ignored-NoGo conditions.

At the same time, the most prominent feature of brain activity during Go/NoGo tasks compared to diverse non-inhibitory tasks is a right-dominant activity in DLPFC [meta-analyses:^3,4,7,9,116^]. Among different response inhibition tasks, several meta-analyses showed that an action-restraint (Go/NoGo task) elicits stronger activation in the right DLPFC and parietal cortex compared to another inhibition process, namely, action cancellation (stop-signal task)^7–9^. Furthermore, the “response uncertainty” tasks induced by the equal probability of Go and NoGo stimuli evoked more activity in the right DLPFC (near the cluster revealed in the current study with coordinates [x = 45 y = 33 z = 28], see Table 2) compared to Go/NoGo and stop-signal tasks with a low probability of inhibitory stimuli (“response override” tasks)^116^.

In meta-analyses by Simmonds et al.^5^ and Criaud and Boulinguez^6^, it was supposed that the increased activity in the right DLPFC observed during Go/NoGo tasks may be due to increased demands caused by working memory and attentional load rather than the inhibition processes per se. The authors explained this by the fact that most of the fMRI studies used complex designs. Therefore, it would be relatively more difficult to identify a NoGo stimulus, which is believed to increase the need for inhibitory control and consequently increase the brain inhibitory activity in NoGo trials. However, this confounding effect was controlled in the current fMRI study because we utilized a simple equiprobable Go/NoGo task.

Another prefrontal structure related to the non-selective response inhibition in the current study was the left IFG (opercular part, AIFO). At the same time, the right IFG is commonly associated with response inhibition^117^. Based on the study of patients with brain damage^118^ and other neuroimaging studies using primarily the stop-signal tasks^117^, it was claimed that the right IFG represents a key node of the response inhibition brain system. Currently, this opinion is under active discussion^119–124^. It is noted that Aron et al.^117^ predominantly relied upon studies using the stop-signal task and did not consider studies using Go/NoGo tasks^121^. Other studies revealed worse performance in the Go/NoGo task when the left (not right) IFG was damaged^120,125^. According to these findings, the left IFG can also participate in inhibitory control during action restraint, which is also confirmed by our results.

Regarding the practically equivalent participation of PMC in both the Go and NoGo trials, it was previously shown to participate in the planning and coordination of actions because its electrical stimulation results in involuntary motor actions^126^. According to Duque et al.^127^, premotor cortex function is associated with inhibition of any premature actions and control of the time of action execution. The authors refer to this brain mechanism as “impulse control”, a concept that is similar to the non-selective response inhibition^18^.

In summary, it can be noted that the selectivity of response inhibition has been studied more extensively to date than its non-selectivity. A considerable amount of research on inhibitory control has used tasks with a low probability of inhibitory stimuli. However, over time, it has become apparent that under several conditions, non-selectivity of response inhibition can, in principle, occur. Studies on non-selectivity of response inhibition typically focus on the possible brain mechanisms for non-selective (“global”) inhibition of motor responses in the settings of interference among multiple concurrent response options^26,27,29,30,32,33,128^. Recently, Criaud et al.^18^ supposed the possibility of involvement of non-selective response inhibition not only for NoGo stimuli but also for Go stimuli. In the current study, the practical equivalence of BOLD signals evoked by equiprobable NoGo and Go stimuli was demonstrated for a number of brain areas associated with response inhibition in an uncertain context, according to our meta-analysis. The present study results proved that response inhibition can act as a non-selective mechanism of action inhibition when the context is uncertain. Thus, one promising area of further research of brain mechanisms of response inhibition (and inhibitory control in general) is the study of the interplay between selective and non-selective inhibition as a function of the contextual uncertainty degree.

## Conclusion

For the first time, combining a meta-analysis and second-level Bayesian analysis yielded results favouring the existence of non-selective response inhibition in equiprobable Go/NoGo task settings. In the present work, selectivity means that inhibition is triggered only by an inhibitory stimulus. The overlap between brain areas previously associated with response inhibition in uncertain context and brain areas demonstrating the practical equivalence of neuronal activity in equiprobable Go and NoGo trials was observed in the right DLPFC, IPL, TPJ, FEF, PMC, and left IFG (AIFO). When a subject was waiting for an equiprobable Go or NoGo stimulus, a non-selective inhibitory control process occurred in both Go trials and NoGo trials in opposition to the model of selective response inhibition. This type of response inhibition prevents the performance of any premature motor actions and operates in a non-selective, “global” mode. Its involvement is favoured by contextual uncertainty caused by the equally probable presentation of Go and NoGo stimuli. Presumably, upon the identification of a Go stimulus, the inhibition is released and the process of action execution is initiated, i.e., it acts as a gating mechanism for accessing a prepared motor program. At the same time, we do not discard the opportunity of involvement of selective inhibitory mechanisms in a less uncertain context. Therefore, future research should address the issue of how brain mechanisms of selective and non-selective response inhibition are inter-related.

### Limitations and further considerations

Generally, it is assumed that the non-selective response inhibition is a relatively early transient process. Therefore, one of the limitations is associated with a low temporal resolution of the fMRI method. Further studies of the early temporal dynamics of neural activity are needed using EEG, MEG, and intracranial recordings in similar uncertain context settings. In particular, evidence for the practical equivalence of early ERP amplitudes (170 ms) revealed previously in equiprobable NoGo and Go trials should be provided in a future research. It is also important to note that we looked for practically equivalent increases in regional brain activity as measured by BOLD-signal. Firstly, it is possible to look for the similarity of local activity patterns by employing multivariate classification techniques in future studies. Secondly, future research should consider how non-selective response inhibition is mediated by interactions between the revealed cortical structures and subcortical structures that form inhibitory and excitatory cortico-striato-thalamo cortical circuits.

Despite the fact that revealed fast reaction times and the LBR increase in NoGo trials evidenced the existence of prepotent response tendency, the low error rate might be a potential limitation of the current study because of the high automaticity of induced behaviour. At the same time, such low commission error rates have been repeatedly reported in several equiprobable Go/NoGo studies^61,71,95–98^. Future research should use objective electrophysiological measures, such as LRPs, subthreshold electromyographic activity, and motor evoked potentials elicited by transcranial magnetic stimulation to rigorously demonstrate prepotent motor activity in equiprobable cue-target Go/NoGo task settings.

The other important challenge for the current study and the field in general is the question of segregation of the brain activity elicited by non-selective response inhibition from that elicited by non-inhibitory processes, like attentional or executive processes. One possible solution may be to look for brain activity that is causally related to inhibitory effects at the level of the primary motor cortex, spinal cord, and effector muscles.

## Supplementary Information

Supplementary Information.

## Data Availability

De-identified neuroimaging data, including all individual contrasts, are available upon reasonable request by message to the corresponding author. The use and sharing of these data must comply with ethics approval of the N.P. Bechtereva Institute of the Human Brain of the Russian Academy of Sciences.

## Code availability

The Matlab code used to convert beta values to PSC and SPM12-based *BayInf* toolbox used to perform Bayesian inference are available on Github (https://github.com/Masharipov/Bayesian_inference). Meta-analyses Z-score maps and thresholded maps, Bayesian analysis and conjunction analysis results are available in Neurovault (https://neurovault.org/collections/6009).
